# A Treatise on the Diseases of the Bones

**Published:** 1850-01

**Authors:** 


*A Treatise on the Diseases of the Bones.* By Edward Stanley, F. R. S., President of the Royal College of Surgeons, England, and Surgeon to St. Bartholomews' Hospital. Philadelphia, Lea & Blanchard. pp. 286. 8vo.

Among the reprints of valuable original works, with which the American press has lately favored the profession, few we think, will be more widely sought for than that of Mr. Stanley on the Bones. Coming from high authority, and treating of a subject, the accurate study of which has been long neglected, this treatise will be found to furnish an amount of practical information, that we are sure will be highly appreciated by most practitioners. Although admitting in the abstract, the evils resulting to English authors from the present state of the copy right, we are convinced that the mass of readers in this country, are benefitted by it, and we cannot but believe that the increased circulation thus furnished to works which would be otherwise unknown, must, by an extended reputation, repay the pecuniary loss which writers imagine they suffer, under existing circumstances. Without, however, wishing to lay open a vexed question, we thank the publishers for the present volume, and congratulate the author on the fact, that though his ideas appear in an American dress, they are not saddled with the name of another, who, as editor or annotator, would doubtless have *improved* upon his opinions.

But before noticing more in detail the volume which heads this article, we cannot resist the desire of giving our feeble praise to a previous work of Mr. Stanley on the same class of diseases. We allude to his magnificent colored "Illustrations" of the Diseases of Bones, &c. In such works, Europe is yet far in advance of us, and we cannot but envy our medical friends there, the opportunities which they possess, of issuing plates which are not only most creditable as artistic pieces, but also admirable, as true delineations of appearances in specimens, which the lapse of a few days would otherwise destroy for ever. Selfish as it may seem, we most sincerely wish, that any of our publishers *could* reprint Mr. Stanley's plates, as readily as they have done his treatise; but as they are as yet not equal to this task, we advise every medical librarian to secure an English copy, assuring them that they will find the volume well worth the cost. For the labor and expense evidently bestowed on his illustrations, Mr. Stanley deserves high praise, and in them he has furnished a volume that will well compare with Cruveilhier, Carswell, &c., and one that must extend his reputation to after ages.

The "Treatise," we are informed in the preface, is an independent volume, having no connection with the Illustrations, except in occasional references. The two works are therefore complete in themselves, and each is independent of the other.

In the arrangement of the Treatise, we are presented, first, with a chapter of general considerations, in which are too briefly specified some of the peculiarities of bone, and in which, we were disappointed in not finding a more detailed account of the special anatomy of their structure. In order properly to appreciate pathological changes, the observer must necessarily be correctly acquainted with healthy tissue. But in the bones, the accounts of minute structure are not universally credited, and we much doubt whether most of those who read the Treatise, will not find themselves unable to understand, in its present form, the opinions which are so concisely given throughout most of its pages. Osteology, as a portion of Histology, is a branch of Anatomy only specially taught within a very few years, and as additions are being constantly furnished by the observations of the microscopists, it requires constant reading to prevent their progress from leaving in a false position, those who rely upon the doctrines taught in the schools in former days.

The body of the work is divided into four parts:

Part 1st embraces eight chapters on changes of structure, and constitutes the basis of many of the subsequent observations. It includes Hypertrophy, Atrophy, Neuralgia, and Inflammation and its results, as Enlargement, Expansion of Tissue, Induration, Osseous deposits, Suppuration, Caries, Ulceration, and Necrosis.

In the first pages of this part, the peculiar arrangement, extreme brevity, and also want of accuracy in the signs furnished as characteristic of the diseases referred to, cannot but be apparent to all; whilst the absence of any observations respecting the Periosteum and its diseases, in this portion of the work, struck us as an error. Why the author prefers placing the periosteum last, is not apparent. We therefore imagined that he entertained some special views of its functions, until, in the chapter on Necrosis, we found that his opinions were very much the same as those of other observers.

The chapter on Neuralgia is also far from satisfactory, as at present described. What portions of structure are affected in this neuralgia? Has Mr. Stanley traced nerves from the nutritious foramen throughout the bone? Or does his neuralgia mean a disorder of the internal or external periosteum and its expansions? Two of the three cases cited as examples of neuralgia, arose from injuries which might have acted on these membranes, and as the diagnosis of neuralgia from periostitis and suppuration within the cancellated tissue, is most important in practice, we cannot but regret that the description is not more explicit.

In the chapters on Suppuration, Caries, Ulceration and Necrosis, we at once find the author's forte, as there is a marked difference in his description of such matters of a practical character, as he has seen in disease, and those more immediately connected with minute changes of structure. In the practical surgery of the volume, the reader will be sure to find much to instruct him, but in the special anatomy and physiology, he will be disappointed. Suppuration, Caries, Necrosis, &c., are from the pen of one who has felt and recognised what he describes, and who, therefore, can point out such lines of difference as at once establish the correctness of his views; but the explanations of minute changes of structure, as the production of tumors, morbid changes, or osseous nourishment in healthy bone, might have been left to other hands.

In a disease of common occurrence, as caries, this is especially true. Mr. Stanley at once recognises the characters of the disease, and does not tell us that caries "is an affection analogous to ulceration in the soft parts," but defines it accurately as "the changes which are consequent on chronic suppuration in the cancellous texture of bones, which, in its progress, exhibits the following phenomena—Inflammation extending from the bone to its investing soft parts; these become swollen, thickened and tender, and abscesses are formed in them which contract into fistulous passages leading to the diseased bone. The bone itself is at first very vascular; then its cells become filled with a reddish-brown fluid, apparently a mixture of blood and pus, and occasionally combined with oily particles."

"Absorption of the bone, but chiefly of its animal part, ensues; that which remains is porous, fragile, and of a grey, brown or black color, probably from decomposition of the matter within its cells, to which cause likewise, the fetid odor of the discharge may be ascribed.

"The surface of ulcerated bone is rough and porous, except in instances where its texture has been previously hardened by inflammation. Around the ulcer, from simple inflammation, its texture becomes expanded and hardened; also around the ulcer of bone, from simple inflammation, osseous deposits occur in the periosteum in the forms of tubercles and spines. But the scrofulous or other specific ulcers of bone are not accompanied by these changes, either in the surrounding periosteum or bone."

Without, however, quoting more, we venture the opinion that any reader of this portion of the work will recognise an ulceration of bone analogous to ulceration of soft parts, but soon find that this ulceration is not caries.

The history, &c., of Necrosis occupies near fifty pages of the book, and will, we think, bear a close scrutiny. In it the reader will find one of the best articles that has yet appeared on this interesting disease, and one also that is fully worked up with the investigations of the day. The necessary brevity of this notice must, however, preclude our doing more than state its apparent value. Our remarks also of the remaining portions of the work must be less extended.

Part II. treats of Tumors of Bone, and contains 1st, Tumors of Bone which pulsate, and 2d, Osseous Growths arising in considerable numbers from the skeleton and in the soft tissues. It occupies fifty-two pages, and contains a large amount of valuable observation. Necrosis and these tumors, are decidedly the portions that will most add to the author's reputation.

Part III. contains Rickets, Conditions of Bone designated as Mollities and Fragilitas, Scrofula in Bone, Hard Carcinoma and Melanosis in Bone.

The article on Scrofula is good as far as it goes, though it is but too evident that the excellent monograph of Nelaton on Tubercles in Bone, published at Paris in 1839, has not reached the author; otherwise we think he would have modified some of his descriptions of the progress of the disease.

Part IV. treats of Morbid Growths from the Jaws, Diseases of the Bones of the Spine, and lastly of Diseases of the Periosteum.

The frequent repetition of the operation of Resection of the Jaws at the present day, will doubtless induce many operators to consult Mr. Stanley's article in the first portion of this part; and as they will not fail to learn something from its perusal, we cannot but hope that they may hesitate longer than we fear is often done, before performing a painful operation, and one which too frequently is of but little service to the patient. Mr. Stanley's observations on the importance of operating so as to avoid if possible injuring many branches of the portio dura, will, we think, be recognised as true. The present number of this Journal contains an improvement by Prof. Wm. E. Horner, on the ordinary mode of operating, which seems likely to accomplish this important object.

In thus hurriedly noticing the Treatise on the Bones, it is evident that we can only imperfectly touch upon the more prominent characteristics of the volume. As a treatise, it wants methodical arrangement and often clearness of description, whilst the brevity of many opinions is a decided fault. But it also contains a large amount of useful information, and must prove a valuable addition to the library of every surgeon. The value of what we do find only makes us wish for more. Coming from the pen of one who has such a fine field for verifying his views, we cannot but feel dissatisfied. We would therefore hint that more Anatomical, Physiological, Microscopical, Pathological and Diagnostic details, might be substituted for many of the accounts of cases and opinions of medicinal articles, which would, we believe, enhance the value of the volume. *

